# Ginkgolide B Blocks Vascular Remodeling after Vascular Injury via Regulating Tgf*β*1/Smad Signaling Pathway

**DOI:** 10.1155/2023/8848808

**Published:** 2023-12-13

**Authors:** Quan Wang, Shuai Ni, Li Ling, Siqi Wang, Hanbin Xie, Zhanhong Ren

**Affiliations:** ^1^Hubei University of Science and Technology, Xianning 437100, China; ^2^German Cancer Research Center (DKFZ), Heidelberg 69120, Germany; ^3^School of Pharmacy, Xianning Medical College, Hubei University of Science and Technology, Xianning 437100, China; ^4^Collections Conservation Research Center, Shanghai Natural History Museum (Branch of Shanghai Science and Technology Museum), Shanghai 200041, China

## Abstract

Coronary artery disease (CAD) is the most prevalent cardiovascular disease worldwide, resulting in myocardial infarction (MI) and even sudden death. Following percutaneous coronary intervention (PCI), restenosis caused by vascular remodeling is always formed at the stent implantation site. Here, we show that Ginkgolide B (GB), a naturally occurring terpene lactone, effectively suppresses vascular remodeling and subsequent restenosis in wild-type mice following left carotid artery (LCA) injury. Additional experiments reveal that GB exerts a protective effect on vascular remodeling and further restenosis through modulation of the Tgf*β*1/Smad signaling pathway *in vivo* and in human vascular smooth muscle cells (HVSMAs) but not in human umbilical vein endothelial cells (HUVECs) *in vitro*. Moreover, the beneficial effect of GB is abolished after incubated with pirfenidone (PFD, a drug for idiopathic pulmonary fibrosis, IPF), which can inhibit Tgf*β*1. In Tgf*β*1^−/−^ mice, treatment with pirfenidone capsules and Yinxingneizhi Zhusheye (including Ginkgolide B) fails to improve vascular remodeling and restenosis. In conclusion, our data identify that GB could be a potential novel therapeutic agent to block vessel injury-associated vascular remodeling and further restenosis and show significant repression of Tgf*β*1/Smad signaling pathway.

## 1. Introduction

Coronary artery disease (CAD) is the leading cause of mortality worldwide [1–4]. Coronary heart disease is characterized by the accumulation of atherosclerotic plaques within the coronary artery wall [5, 6]. When these plaques become large, unstable, ruptured, or erosive, it will lead to myocardial infarction (MI) and sudden death [5, 6]. Percutaneous coronary intervention (PCI) is the most common treatment strategy for coronary heart disease [7]. Millions of people in worldwide undergo PCI procedures every year. Vascular stent interventional therapy is the main clinical treatment method for cardiovascular diseases, as it causes less trauma and allows for faster recovery [8]. However, vascular remodeling-induced restenosis and stent thrombosis have become the most important clinical issues after PCI [9, 10].

Endothelial cells inevitably experience near-complete damage and loss after stent implantation [11, 12]. Local blood flow disorder and complex changes in shear stress occur after stent implantation, which alters the phenotype of endothelial cells from static to inflammatory and increases the possibility of thrombosis [12]. When the intima is severely injured, the smooth muscle cells within the arterial media can migrate into the intima. Neointimal formation and vascular restenosis are caused by the migration, proliferation, and production of connective tissue by vascular smooth muscle cells (VSMCs) [13]. Transforming growth factor-*β* (Tgf*β*) plays a critical role in biological processes, including the control of cell proliferation and differentiation, the regulation of tissue repair and extracellular matrix accumulation, and the regulation of immune and inflammatory responses [14]. Previously, studies demonstrated that Tgf*β* has been found to possess proinflammatory, profibrotic, and antiendothelial regeneration abilities [15–17].

Ginkgolide B (GB), a naturally occurring terpene lactone, is an active component extracted from the leaves of Ginkgo biloba [18]. Previous studies have demonstrated that GB possesses numerous pharmacological properties, including antifibrosis, anti-inflammatory, and antithrombosis activities [19–21]. A mixture of Ginkgolide A, B, and C has undergone phase III clinical studies and has become a preferred natural drug for the treatment of cardiovascular and cerebrovascular diseases [22–25]. GB exhibits stronger inhibition of platelet-activating factor (PAF) [26]. PAF plays an important role in the process of promoting thrombosis, as it has a unique and strong ability to promote platelet aggregation and release [27]. Therefore, whether GB can improve vascular remodeling to reduce vessel thrombosis and restenosis and its possible regulatory mechanisms remain unknown.

The objective of this study was to investigate the function of GB in vascular injury. First, we sought to demonstrate whether treatment with GB could improve vascular remodeling in WT mice following vascular injury. Second, we aimed to identify the underlying mechanism of GB treatment function. Third, we aimed to determine the specific cell lines influenced by GB. Lastly, we aimed to expand the indications of some potential clinical applications of GB.

## 2. Materials and Methods

### 2.1. Reagents

Ginkgolide B (purity: 98% by HPLC) was supplied by the National Institutes for the Control of Pharmaceutical and Biological Products (Beijing, China). Pirfenidone (Cat number S2907) was obtained from Selleck (Shanghai, China). The drug of Yinxingneizhi Zhusheye (including Ginkgolide B) was obtained from Chengdu Baiyu Pharmaceutical Company. The drug of pirfenidone capsules was purchased from Beijing Continent Pharmaceuticals Company. Tgf*β*1 recombinant protein (Cat number H00007040-P01) was bought from Amyjet Scientific (Wuhan, China). The cell count kit-8 (Cat number CA1210) was bought from Solarbio Life Sciences (Shanghai, China). Rabbit monoclonal to Tgf*β*1 (Cat number ab179695) was obtained from Abcam (CA, USA). Phospho-Smad2 (Ser465/467)/Smad3 (Ser423/425) (D27F4) Rabbit mAb (Cat number #8828) was applied by Cell Signaling Technology. GAPDH monoclonal antibody (Cat number 60004-1-Ig) was bought from Proteintech (Rosemont, USA).

### 2.2. Animals and Procedures

Wild-type C57BL/6 J mice were purchased from Liaoning Changsheng biotechnology (Changchun, China). This study was approved by the Animal Ethics Committee of Hubei University of Science and Technology (No. 202204051), China. The homozygous Tgf*β*1^−/−^ knockout (KO) mice were bought from Cyagen (Suzhou, China). The eight-week-old mice were used to establish the vascular injury model as reported [28]. In brief, mice were anesthetized with 3% pelltobarbitalum natricum (0.1 ml/10 g body weight) and then transferred to a warmer pad. Firstly, after depilation of the neck, the skin was cut along the midline of the neck using scissors, and the left carotid artery was exposed by blunt separation with forceps. Subsequently, the left carotid artery and accompanying nerve were bluntly separated under a microscope using microforceps, the proximal end was clamped with a vascular clip, the internal carotid artery was ligated with a 5-0 slip-tie, and the distal end of the external carotid artery was ligated with a 5-0 thread. A metal wire was then inserted from the distal end of the external carotid artery to the left carotid artery and repeated three times to damage the endothelial cells. After that, the metal wire was withdrawn, the bifurcation of the external carotid artery and the internal carotid artery was ligated with 5-0 suture, the slipknot of the internal carotid artery and the vascular clip of the left carotid aorta was released, and the wound was sutured.

The mice were maintained in a humidity range of (50 ± 10)%, temperature of (23 ± 2) °C, and a 12-hour light/dark cycle with free access to food and water. The WT mice were randomly distributed into two groups (*n* = 12 each group) and treated for 28 days: (1) vehicle (1% DMSO and 5% carboxymethylcellulose sodium; intragastric administration) treatment group—LCA was surgery group, and RCA was corresponding control group; (2) GB (30 mg/kg/day; intragastric administration) treatment group—LCA was surgery group, and RCA was corresponding control group. The Tgf*β*1^−/−^ mice were divided into three groups (*n* = 8 each group) and treated for 28 days: (1) vehicle (1% DMSO and 5% carboxymethylcellulose sodium; intragastric administration) treatment group—LCA was surgery group, and RCA was corresponding control group; (2) pirfenidone capsules (72 mg/kg/day, calculated from the body surface area of human and mouse; intragastric administration) treatment group—LCA was surgery group, and RCA was corresponding control group; (3) Yinxingneizhi Zhusheye (30 mg/kg/day; intraperitoneal injection) treatment group—LCA was surgery group, and RCA was corresponding control group.

### 2.3. Echocardiography

Echocardiography was performed using Vevo 2100 High Resolution Micro Ultrasound System (Visual Sonics, Toronto, Canada) by a blinded researcher as reported previously [29]. In brief, mice were anesthetized with 3% pelltobarbitalum natricum (0.1 ml/10 g body weight) and then transferred to an operating platform to remove the neck coat. Next, the Doppler angle was adjusted and optimized, and then, the probe was used to measure blood flow in LCA and RCA.

### 2.4. Histology

The method of histology was previously reported [29]. The isolated LCA and RCA obtained from the mice in all experimental groups were fixated in 4% paraformaldehyde and cryosectioned into a 4 *μ*m section. Subsequently, the sections were stained with hematoxylin and eosin (H&E) and Masson's trichrome. Images were collected using a light microscope (Nikon Corporation), and morphometric analysis was performed using ImageJ software.

### 2.5. Cell Culture

The human umbilical vein endothelial cells (HUVECs) were purchased from Mingzhou company (Cat number MZ-5180). The human aorta vascular smooth muscle cells (HVSMCs) were obtained from Mingzhou company (Cat number MZ-2709). The cells were incubated with 10% fetal bovine serum (Gibco Life Technologies, Cat number 10099) and penicillin-streptomycin (Beyotime, Cat number ST488). Subsequently, the cells were cultured in a cell incubator at 37°C with 5% CO_2_.

### 2.6. Quantitative Real-Time RT-PCR Analysis

The method of histology was previously reported [30]. In brief, the total RNA was extracted from tissues and cells using Trizol (Bolaz, Cat number RE0101, Nanjing, China) and reverse transcribed into cDNA by a cDNA Synthesis Kit (Bolaz, Cat number QP0612, Nanjing, China). Quantitative real-time PCR analysis was completed using the SYBR green qPCR mix (low Rox) (Bolaz, Cat number QP0602, Nanjing, China) on 7900 HT Fast Real-Time PCR system (ABI).

The primers are the following:

Human *Mcp1*: monocyte chemoattractant protein 1 (NM_002982.4)

Forward primer: CTCGCTCAGCCAGATGCAAT

Reverse primer: TTGGGTTTGCTTGTCCAGGT

Mouse *Mcp1*: monocyte chemoattractant protein 1 (NM_011333.3)

Forward primer: CACTCACCTGCTGCTACTCA

Reverse primer: TGAGCTTGGTGACAAAAACTACAG

Human *Cd68*: cluster of differentiation 68 (NM_001040059.2)

Forward primer: GGCTACTGGCAGAGAGCAC

Reverse primer: CTAGTGGTGGCAGGACTGTG

Mouse *Cd68*: cluster of differentiation 68 (NM_001291058.1)

Forward primer: AGGACCGCTTATAGCCCAAG

Reverse primer: TGCCATTTGTGGTGGGAGAA

Human *α-Sma: α*-smooth muscle actin (NM_001141945.3)

Forward primer: CTCAACGTGGAGCGCAGT

Reverse primer: GCTTCACAGGATTCCCGTCT

Mouse *α-Sma: α*-smooth muscle actin (NM_007392.3)

Forward primer: CTTCGTGACTACTGCCGAGC

Reverse primer: AGGTGGTTTCGTGGATGCC

Human *Tgfβ1*: transforming growth factor beta 1 (NM_000660.7)

Forward primer: TACCTGAACCCGTGTTGCTC

Reverse primer: CCGGTAGTGAACCCGTTGAT

Mouse *Tgfβ1*: transforming growth factor beta 1 (NM_011577.2)

Forward primer: ACGTGGAAATCAACGGGATCA

Reverse primer: GTTGGTATCCAGGGCTCTCC

### 2.7. Western Blot

The method was followed as reported [31]. The proteins were extracted from the tissues (four LCA or RCA samples were pooled into one sample) and cells using radio immunoprecipitation assay lysis buffer (Beyotime, Cat number P0013B) with protease and phosphatase inhibitor cocktail (Beyotime, Cat number P1048). The samples were then separated using 10% sodium dodecyl sulfate polyacrylamide gel electrophoresis (SDS-PAGE) and transferred to polyvinylidene difluoride (PVDF) membranes (Millipore). Subsequently, the membranes were blocked with blocking buffer (Bolaz, Cat number PP1205, Nanjing, China) for 2 hours at room temperature and incubated with primary antibodies overnight at 4°C. The next day, the membranes were washed with phosphate-buffered saline (PBS) three times for 5 minutes and incubated with corresponding secondary antibodies for 2 hours at room temperature. The pictures were obtained from Quantity One (Bio-Rad) and analyzed by ImageJ software.

### 2.8. Enzyme-Linked Immunosorbent Assays (ELISA)

The concentration of Tgf*β*1, Mcp1, and *α*-Sma in animal serum and cell culture supernatant were detected by ELISA kit according to the manufacturer's instructions. The ELISA assay kits used were as follows: human transforming growth factor-*β*1 (Tgf*β*1; Cat number JL10706) and mouse transforming growth factor-*β*1 (Tgf*β*1; Cat number JL12223), mouse *α*-smooth muscle actin (*α*-Sma; JL20208), and monocyte chemoattractant protein 1 (Mcp1; Cat number JL20304), all supplied by Jianglai Biology (Shanghai, China).

### 2.9. Cell Viability Assays

The cells were seeded into 96-well plates at a density of 2 ~ 3 10^3^ cells per well and cultured for 36 hours, followed by an assessment of cell viability using a Cell Kit-8 according to the manufacturer's protocols.

### 2.10. Molecular Docking

The method was followed as reported [32]. Docking of Ginkgolide B and Tgf*β*1 was explored using the MOE database via molecular operating environment (MOE v2019.0101, Chemical Computing Group Inc., Montreal, QC, Canada). Following the Protein Data Bank (RCSB Protein Data Bank-RCSB PDB, http://www.pdb.org/), we got the Tgf*β*1 protein structure data with structure ID 3TZM.

### 2.11. Statistical Analysis

The data were analyzed using GraphPad Prism 8.0.1 and expressed as mean ± standard error of mean (SEM). The data were judged to be statistically significant when *P* was <0.05. Two-tailed Student's *t*-tests or one-way analysis of variance (ANOVA) was used to analyze data.

## 3. Results

### 3.1. Ginkgolide B Improves Vascular Remodeling and Inflammation after Vessel Injury

**Figure 1:**
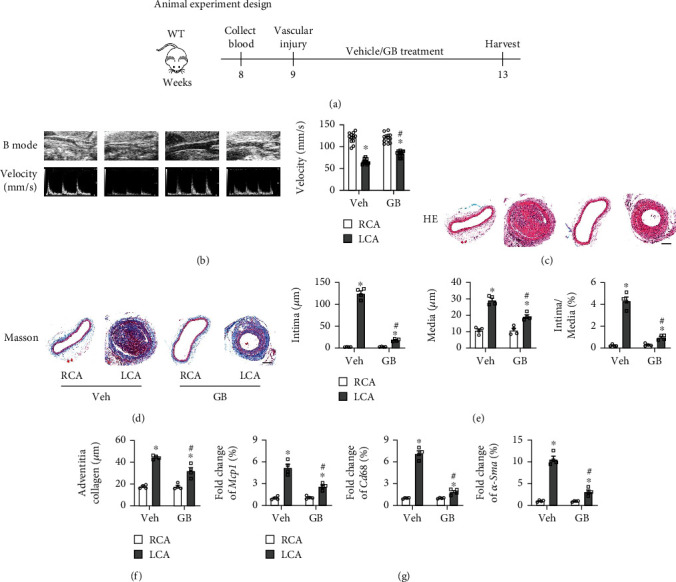
Ginkgolide B improves vascular remodeling and inflammation after vessel injury. (a) The schedule of animal experiment design. Collect blood from orbital venous plexus. (b) The detection of blood flow velocity by the Doppler echocardiography (*n* = 12 each group). The histological analysis performed using H&E staining (c) and the corresponding quantification (e) and Masson's staining (d) and the corresponding quantification (f) (scar bar: 50 *μ*m; *n* = 4 each group). (g) The mRNA expression of *Mcp1* (monocyte chemoattractant protein 1), *Cd68* (cluster of differentiation 68), and *α-Sma* (*α*-smooth muscle actin) in tissues (*n* = 4 each group). All data are presented as mean ± SEM. ^∗^*P* < 0.05 versus RCA treatment with vehicle; ^#^*P* < 0.05 versus LCA treatment with vehicle.

Clinically, after PCI procedures are performed to treat blocked blood vessels in patients, severe restenosis often occurs at the site of stent implantation due to vascular remodeling one year postprocedures, representing a significant threat to the patient's life safety. To explore the function of Ginkgolide B (GB) in vascular remodeling and its potential impact on restenosis formation, a wire injury model was established in murine left carotid artery (LCA), which the right carotid artery (RCA) of the same murine served as a control group (Figure 1(a)). After treatment with GB (30 mg/kg/day) by intragastric administration for 28 days, a serious of detections were performed. The Doppler echocardiography revealed a significantly decreased blood flow velocity in the LCA compared with the corresponding RCA (Figure 1(b)). After GB treatment, the blood flow velocity was markedly increased in wire-injured LCA than vehicle treatment group (Figure 1(b)). GB treatment did not significantly affect RCA blood flow nor did vehicle treatment (Figure 1(b)). H&E staining revealed that the carotid artery walls of LCA were markedly thickened, and the intimal thickness and medial thickness were significantly increased compared to RCA after LCA injury, indicating vessel remodeling (Figures 1(c) and 1(e)). However, the vessel remodeling phenotypes of LCA were significantly improved after treatment with GB (Figures 1(c) and 1(e)). Furthermore, Masson's staining demonstrated that the fibrosis is significantly upregulated in the LCA compared to the RCA, and GB treatment significantly downregulated fibrosis in the RCA compared to vehicle treatment of the LCA (Figures 1(d) and 1(f)). Additionally, the mRNA expression levels of *Mcp1* (monocyte chemoattractant protein 1, marker for inflammation), *Cd68* (cluster of differentiation 68, marker for macrophage infiltration), and *α-Sma* (*α*-Smooth muscle actin, marker for VSMC proliferation) were significantly upregulated in LCA compared to RCA (Figure 1(g)). Moreover, mice treated with GB exhibited a significant inhibition in the expression of *Mcp1*, *Cd68*, and *α-Sma* in the LCA (Figure 1(g)). These data revealed that fibrosis, inflammation, infiltration, and VSMC proliferation were significantly increased in LCA compared to RCA. In conclusion, GB treatment plays a critical role in attenuating vascular modeling and further restenosis induced by vessel injury.

### 3.2. Ginkgolide B Plays a Protective Role in Vascular Injury by Tgf*β*1/Smad Signal Pathway

**Figure 2:**
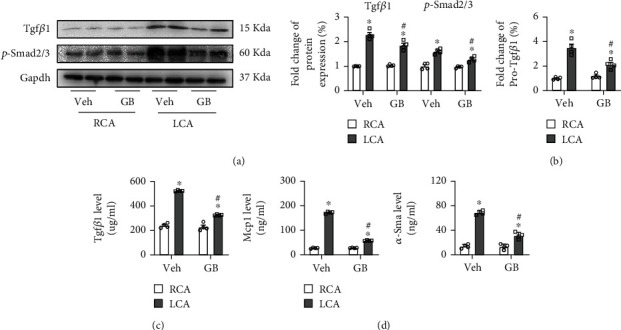
Ginkgolide B plays a protective role in vascular injury by the Tgf*β*1/Smad signal pathway. (a) The protein level detection of Tgf*β*1 and p-Smad2/3 and quantification *in vivo*. (b) The mRNA level detection of Pro-Tgf*β*1 *in vivo*. The ELISA detection of Tgf*β*1 (c), Mcp1 (d), and *α*-Sma (d) in serum. All data (*n* = 4 each group) are presented as mean ± SEM. ^∗^*P* < 0.05 versus RCA treatment with vehicle; ^#^*P* < 0.05 versus LCA treatment with vehicle.

Previously, studies demonstrated that Tgf*β*1 plays a key role in fibrosis, inflammation, and VSMC proliferation [19, 20, 33, 34]. We hypothesized that the protective function of GB in vascular remodeling is performed by regulating the Tgf*β*1 signal pathway. Western blotting showed that the protein levels of Tgf*β*1 and phosphorylated Smad2/3 (p-Smad2/3) were notably increased in murine-injured LCA compared with murine RCA (Figure 2(a)). Additionally, GB treatment significantly reduced Tgf*β*1 and p-Smad2/3 expressions at the protein level compared with vehicle treatment in the LCA group (Figure 2(a)). Similar to the results at the protein level, the mRNA expression of Tgf*β*1 was markedly upregulated after LCA vessel injury, and this upregulation was repressed by GB treatment (Figure 2(b)). Furthermore, the levels of Tgf*β*1 in mouse serum were found to have similar sequences (Figure 2(c)). When we detected the levels of Mcp1 and *α*-Sma in mouse serum, we found that these were greatly elevated in the LCA and were dramatically downregulated after GB treatment (Figure 2(d)). Taken together, these findings demonstrate that GB exhibits a protective role in vascular remodeling by Tgf*β*1/Smad signal pathway.

### 3.3. Ginkgolide B Has No Influence of Tgf*β*1/Smad Signal Pathway in Human Umbilical Vein Endothelial Cells (HUVECs)

**Figure 3:**
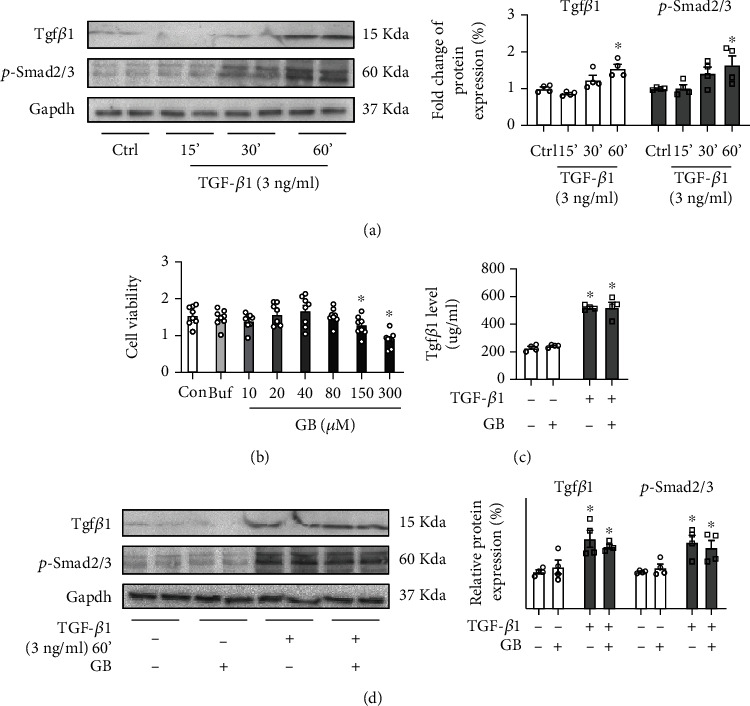
Ginkgolide B has no influence of Tgf*β*1/Smad signal pathway in human umbilical vein endothelial cells (HUVECs). (a) The western blot analysis for Tgf*β*1 and p-Smad2/3 in HUVECs stimulated with 3 ng/ml TGF-*β*1 for 15 min, 30 min, and 60 min (*n* = 4 each group). (b) The cell viability of HUVECs incubated with different concentrations of GB was detected by CCK8 (*n* = 8 each group). (c) The ELISA detection of Tgf*β*1 in cell culture supernatant (*n* = 4 each group). (d) The western blot analysis for Tgf*β*1 and p-Smad2/3 in HUVECs stimulated with 3 ng/ml TGF-*β*1 for 60 min and then treated with GB for 2 hours (*n* = 4 each group). All data are presented as mean ± SEM. ^∗^*P* < 0.05 versus control.

Vascular remodeling and further restenosis are primarily caused by the proliferation and migration of endothelial cells or VSMCs [35–40]. To further confirm the underlying mechanism of GB *in vitro*, a Tgf*β*1-stimulated HUVEC model was first established. Previously, articles have demonstrated that 3 ng/ml TGF-*β*1 could significantly upregulate the expression of Tgf*β*1 *in vitro* [41]. After being incubated with 3 ng/ml TGF-*β*1 for 15 min, 30 min, and 60 min in HUVECs, the protein levels of Tgf*β*1 and p-Smad2/3 were detected by western blot (Figure 3(a)). The results showed that Tgf*β*1 and p-Smad2/3 were obviously increased after incubated for 60 min compared with the control group (Figure 3(a)). Then, the cytotoxic effects of GB at different doses on HUVECs were assessed using the cell counting kit-8. GB at the low concentrations (10, 20, 40, and 80 *μ*M) had no significant cytotoxic effect on HUVECs, while 150 and 300 *μ*M of GB resulted in a sharp reduction in cell viability compared with the control group, indicating that a low concentration of 40 *μ*M GB was suitable for performing other experiments (Figure 3(b)). Then, HUVECs were incubated with 3 ng/ml TGF-*β*1 for 60 min, and GB were added to treat HUVECs for 2 hours. We also measured the level of Tgf*β*1 in the supernatant cell culture, which also demonstrated that GB had no effect on adjusting the Tgf*β*1/Smad signal pathway (Figure 3(c)). Additionally, the western blot revealed that GB could not regulate the Tgf*β*1/Smad signal pathway in HUVECs regardless of whether they were stimulated with TGF-*β*1 (Figure 3(d)). These findings indicated that GB has no influence on the Tgf*β*1/Smad signal pathway in HUVECs.

### 3.4. Ginkgolide B Regulates Tgf*β*1/Smad Signal Pathway in Human Vascular Smooth Muscle Cells (HVSMAs)

**Figure 4:**
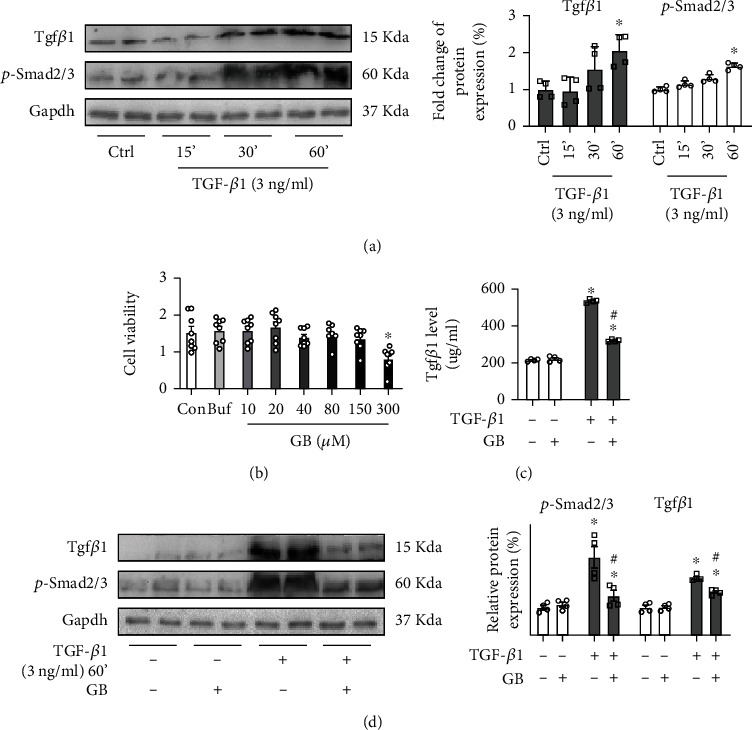
Ginkgolide B regulates the Tgf*β*1/Smad signal pathway in human vascular smooth muscle cells (HVSMAs). (a) The western blot analysis for Tgf*β*1 and p-Smad2/3 in HVSMAs stimulated with 3 ng/ml TGF-*β*1 for 15 min, 30 min, and 60 min (*n* = 4 each group). (b) The cell viability of HVSMAs incubated with different concentrations of GB was detected by CCK8 (*n* = 8 each group). (c) The ELISA detection of Tgf*β*1 in cell culture supernatant (*n* = 4 each group). (d) The western blot analysis for Tgf*β*1 and p-Smad2/3 in HVSMAs stimulated with 3 ng/ml TGF-*β*1 for 60 min and then treated with GB for 2 hours (*n* = 4 each group). All data are presented as mean ± SEM. ^∗^*P* < 0.05 versus control.

To further determine whether GB plays a protective role in HVSMCs through the Tgf*β*1/Smad signal pathway, TGF-*β*1 concentration-dependent experiments and HVSMA viability assays were conducted (Figures 4(a) and 4(b)). These results repeatedly showed that 3 ng/ml TGF-*β*1 incubated for 60 min and 40 *μ*M GB treatment were optimal (Figures 4(a) and 4(b)). HVSMAs were stimulated with 3 ng/ml TGF-*β*1 for 60 min and then treated with GB for 2 hours. The Tgf*β*1 level in supernatant cell cultures was significantly repressed after GB treatment (Figure 3(c)). Additionally, the western blot revealed that the protein levels of Tgf*β*1 and p-Smad2/3 were obviously decreased by GB treatment in HVSMCs (Figure 3(d)). Collectively, these findings suggest that GB exerts a protective effect on HVSMAs by inhibiting the Tgf*β*1/Smad signal pathway.

### 3.5. The Protective Role of Ginkgolide B Is Abolished after Incubated with Tgf*β*1 Inhibitor in HVSMAs

**Figure 5:**
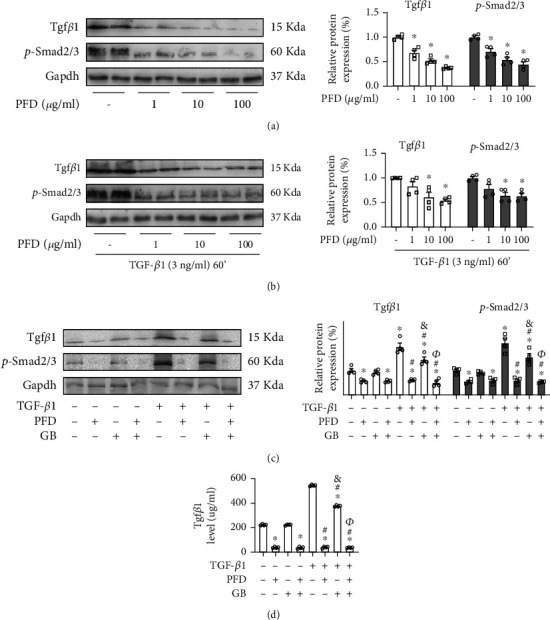
The protective role of Ginkgolide B is abolished after incubated with Tgf*β*1 inhibitor in HVSMAs. (a) The western blot analysis for Tgf*β*1 and p-Smad2/3 stimulated with different concentration of pirfenidone (PFD, Tgf*β*1 inhibitor). (b) The western blot analysis for Tgf*β*1 and p-Smad2/3 stimulated with 3 ng/ml TGF-*β*1 for 60 min and then treated with different concentration of pirfenidone. (c) The western blot analysis for Tgf*β*1 and p-Smad2/3 stimulated with 3 ng/ml TGF-*β*1 for 60 min and then treated with GB or pirfenidone for 2 hours. (d) The ELISA detection of Tgf*β*1 in cell culture supernatant. All data (*n* = 4 each group) are presented as mean ± SEM. ^∗^*P* < 0.05 versus control; ^#^*P* < 0.05 versus treatment with TGF-*β*1; ^&^*P* < 0.05 versus treatment with TGF-*β*1 and pirfenidone; ^*Φ*^*P* < 0.05 versus treatment with TGF-*β*1 and GB.

To further confirm the inhibitory effect of GB on Tgf*β*1, pirfenidone (PFD), a Tgf*β*1 inhibitor, was applied to verify the function of GB. Firstly, HVSMAs were incubated with different concentrations of pirfenidone for 2 hours to identify a feasible concentration range (Figure 5(a)). The western blot revealed that the expression of Tgf*β*1 was significantly repressed by pirfenidone, and p-Smad2/3 was also obviously decreased (Figure 5(a)). Then, we further detected the suppressive function of pirfenidone on TGF-*β*1 stimulated (Figure 5(b)). The results showed that after being stimulated with 3 ng/ml TGF-*β*1 for 60 min, pirfenidone concentrations greater than 1 *μ*g/ml could play a repressive role in Tgf*β*1 and downstream p-Smad2/3 at the protein level (Figure 5(b)). In addition, after being incubated with 3 ng/ml TGF-*β*1 for 60 min, 1 *μ*g/ml pirfenidone and 40 *μ*M GB were applied to treat HVSMAs for 2 hours (Figure 5(c)). We found that treatment with pirfenidone significantly decreased Tgf*β*1 and subsequent p-Smad2/3 levels, regardless of whether the cells were stimulated with TGF-*β*1, but treatment with GB did not affect these levels when no TGF-*β*1 stimulation was present (Figure 5(c)). Following stimulation with TGF-*β*1, treatment with either pirfenidone or GB alone repressed the Tgf*β*1/Smad signaling pathway, but the inhibitory ability of GB was obviously weaker than that of pirfenidone (Figure 5(c)). However, when treated with a combination of pirfenidone and GB, the restrictive ability of the Tgf*β*1/Smad signaling pathway was approximately equivalent to that of a single treatment with pirfenidone alone, indicating that the function of GB on the Tgf*β*1/Smad signaling pathway was replaced by the stronger inhibitor pirfenidone (Figure 5(c)). Interestingly, the detection of Tgf*β*1 in supernatant cell cultures was consistent with the protein expression outcomes (Figure 5(d)). In conclusion, these results revealed that the suppressive function of GB on the Tgf*β*1/Smad signaling pathway is abolished by treatment with the Tgf*β*1 inhibitor pirfenidone.

### 3.6. The Protective Function of Ginkgolide B in Vessel Remodeling and Inflammation Caused by Vascular Injury Is Vanished in Tgf*β*1 Knockout Mice

**Figure 6:**
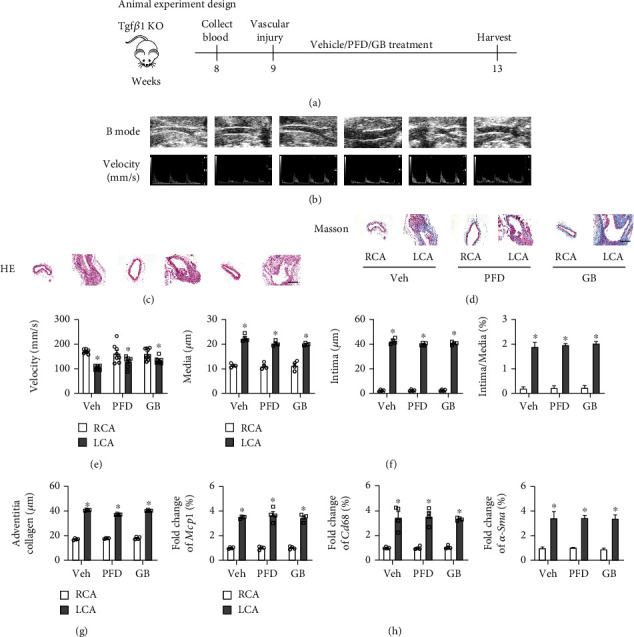
The protective function of Ginkgolide B in vessel remodeling and inflammation caused by vascular injury is vanished in Tgf*β*1 knockout mice. The mice were treated with pirfenidone capsules (presenting as PFD) and Yinxingneizhi Zhusheye (including Ginkgolide B) (presenting as GB). (a) The schedule of animal experiment design. Collect blood from orbital venous plexus. (b) The detection of blood flow velocity by the Doppler echocardiography and quantification (e) (*n* = 8 each group). The histological analysis performed using H&E staining (c) and the corresponding quantification (f) and Masson's staining (d) and the corresponding quantification (g) (scar bar: 50 *μ*m; *n* = 4 each group). (h) The mRNA expression of *Mcp1*, *Cd68*, and *α-Sma* in tissues (*n* = 4 each group). All data are presented as mean ± SEM. ^∗^*P* < 0.05 versus RCA treatment with vehicle.

Conclusively, the beneficial function of GB *in vivo* (wild-type mice) and *in vitro* (HVSMAs) was both confirmed by regulating the Tgf*β*1/Smad signaling pathway. The drug pirfenidone capsules used to treat idiopathic pulmonary fibrosis by decreasing the expression of Tgf*β*1 and the drug Yinxingneizhi Zhusheye (including Ginkgolide B) were used to identify whether GB played a protective role in vascular remodeling by regulating the Tgf*β*1/Smad signaling pathway. Hence, the Tgf*β*1 knockout mice (Tgf*β*1^−/−^) were subjected to LCA injury by wire and treated with Yinxingneizhi Zhusheye (30 mg/kg/day; intraperitoneal injection) and pirfenidone capsules (72 mg/kg/day; intragastric administration) for 28 days (Figure 6(a)). The Doppler echocardiography showed that in Tgf*β*1^−/−^ mice, the blood flow velocity in LCA was significantly decreased after vascular injury, and treatment with pirfenidone capsules and Yinxingneizhi Zhusheye did not improve blood flow velocity (Figures 6(b) and 6(e)). H&E staining revealed that the vessel remodeling was also present in the LCA of Tgf*β*1^−/−^ mice, indicating that the neointimal formation is not solely dependent on the Tgf*β*1/Smad signaling pathway (Figure 6(c)), but the vessel was not entire blocked compared to WT mice (Figure 1(c)). The indices of vessel remodeling were quantified, showing that the carotid artery walls of the LCA were markedly thickened, and the intimal thickness and medial thickness were obviously increased after LCA injury in Tgf*β*1^−/−^ mice (Figures 6(c) and 6(f)). Notably, there was no obvious improvement in vessel remodeling in the LCA of Tgf*β*1^−/−^ mice treated with pirfenidone capsules and Yinxingneizhi Zhusheye (Figures 6(c) and 6(f)). Moreover, aortic collagen deposition was detected by Masson's staining. The results showed that the collagen deposition in the LCA was significantly enhanced in Tgf*β*1^−/−^ mice after vascular injury (Figures 6(d) and 6(g)). Similarly, pirfenidone capsules and Yinxingneizhi Zhusheye were unable to effectively repress collagen formation (Figures 6(d) and 6(g)). The mRNA expressions of *Mcp1*, *Cd68*, and *α-Sma* in the LCA of Tgf*β*1^−/−^ mice were both significantly increased compared to the corresponding group (Figure 6(h)). The inflammation and macrophage infiltration in the LCA of Tgf*β*1^−/−^ mice could not be attenuated by pirfenidone capsules and Yinxingneizhi Zhusheye (Figure 6(h)). Taken together, these results demonstrated that the protective effect of GB on vascular remodeling and further restenosis is abolished after reducing the Tgf*β*1/Smad signaling pathway.

### 3.7. The Molecular Docking of Ginkgolide B and Tgf*β*1

**Figure 7:**
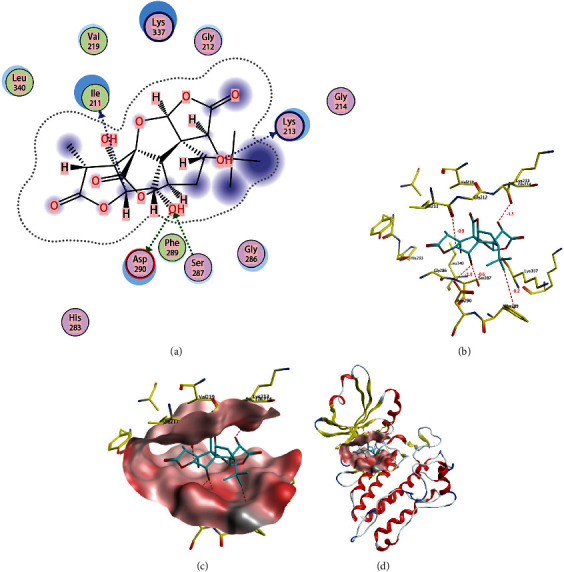
The molecular docking of Ginkgolide B and Tgf*β*1. (a) 2D map. (b–d) The different form of 3D map.

Although these results demonstrated that Ginkgolide B could regulate Tgf*β*1, the underlying regulation mechanism is unknown. To further resolve the question, we suspected that Ginkgolide B could bind with Tgf*β*1 to competitively reduce the bind of Tgf*β*1 and its receptor. According to the molecular docking results, Tgf*β*1 has four sites that can form hydrogen bonds with Ginkgolide B. These four sites are ASP 290, LYS 213, ILE 21, and SER 287, with corresponding distances and energies (kcal/mol) of 2.67 and -1.9, 2.67 and -1.5, 3.49 and -0.9, and 2.99 and -0.6, respectively (Figure 7). These results indicate that there is a certain binding ability between Tgf*β*1 and Ginkgolide B.

## 4. Discussion

**Figure 8:**
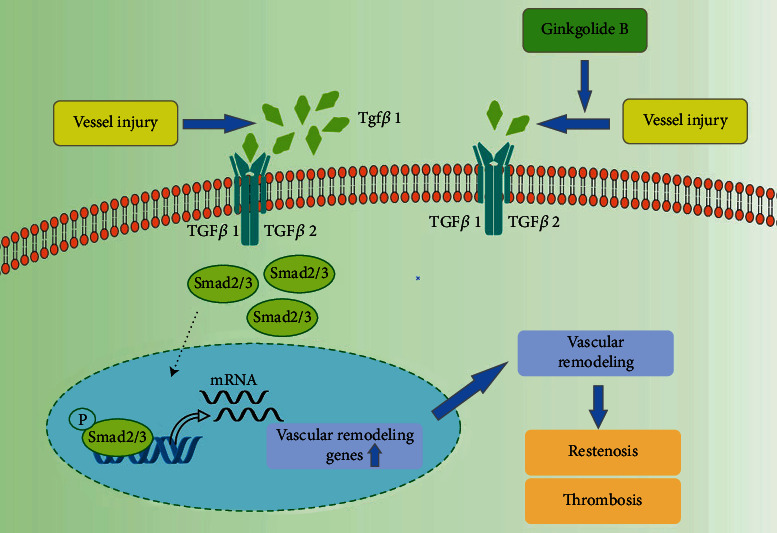
Molecular mechanism involved in vascular remodeling. Vessel injury as in the case of PCI causes Tgf*β*1, activates Smad2/3 phosphorylation, and contributes to the transcription of genes taken part in vascular remodeling. Ginkgolide B represses the production of Tgf*β*1, further reduces Smad2/3 phosphorylation, and contributes to the decline transcription of genes taken part in vascular remodeling, which could decrease restenosis and thrombosis.

In this study, we demonstrated that the GB treatment downregulates the Tgf*β*1/Smad signaling pathway, exerting a protective role in vascular remodeling and further restenosis (Figure 8). Specifically, we found that vascular remodeling and restenosis are severe after LCA injury by wire but are obviously improved by GB treatment (Figure 1). The experiments *in vivo* showed that the beneficial improvement of GB on vascular remodeling and restenosis is due to its regulation of the Tgf*β*1/Smad signaling pathway (Figures 1 and 2). Next, cell experiments demonstrated that GB could repress the Tgf*β*1/Smad signaling pathway in HVSMCs but not HUVECs (Figures 3 and 4). When incubated with pirfenidone, an inhibitor of Tgf*β*1, the function of GB is abolished (Figure 5). Moreover, in Tgf*β*1^−/−^ mice, pirfenidone capsules and Yinxingneizhi Zhusheye do not exert a beneficial effect on vascular remodeling and restenosis after LCA injury (Figure 6).

In the next 30 years, the number of people beyond 65 will double, and chronic inflammation is one of the important causes of natural aging [42]. Vascular remodeling induced by chronic inflammation is particularly important for the body, as it can lead to various vascular diseases such as vascular remodeling, embolism, and atherosclerosis [42]. In this study, we demonstrated Ginkgolide B as a novel candidate drug of vascular inflammation and remodeling associated with vessel injury. Our data showed that GB could significantly suppress vascular inflammation and remodeling by regulating the Tgf*β*1/Smad signaling pathway in HVSMCs. We propose that Tgf *β*1 has a promoting effect on vascular inflammation and remodeling, while GB is a natural Tgf*β*1 antagonist, which can reduce vascular inflammation and remodeling caused by vascular injury.

Due to the influence of bad diet, lifestyle, or heredity, blood vessel stenosis or partial heart disease can lead to insufficient blood supply [43]. At present, the clinical application of stent implantation primarily focuses on addressing severely occluded vessels and restoring normal blood supply. Although modern stents have been upgraded from metal stents to drug elution stents, including antiplatelet aggregation drugs such as aspirin and other drugs to reduce thrombosis and restenosis, the possibility of restenosis still exists [44]. In addition, patients still need to take antiplatelet drugs such as aspirin and clopidogrel for a long time, but the side effects can be serious. These include allergic reactions, gastrointestinal discomfort, and hypersensitivity reactions, which can lead to poor compliance with medication. In this study, we found that the existing clinical drugs, ginkgolide injection and pirfenidone capsule, can significantly reduce vascular inflammation and vascular remodeling caused by vascular injury. Our study provides some basic support for expanding the indications of Yinxingneizhi Zhusheye and pirfenidone capsule in clinical practice.

Interestingly, the study found that GB could regulate the Tgf*β*1/Smad signal pathway in HVSMCs, not in HUVECs. Numerous studies have shown that the LAP-Tgf*β*1 in the extracellular matrix cleave to mature Tgf*β*1 under certain stimuli and bind to Tgf*β*1 receptors on the membrane, leading to phosphorylation of Smad2/3 in the cytoplasm and nuclear entry, promoting transcription of inflammatory factors [29, 45]. GB acts on smooth muscle cells but not endothelial cells. We speculate that this is due to the different receptors present on the cell membranes of the two cells. Da et al. found that AGGF1 acts on the integrin *α*7 receptor on the smooth muscle cell membrane, enhancing the interaction between integrin *α*7 and Tgf*β*1, thereby inhibiting cleavage of LAP-Tgf*β*1 and production of mature Tgf*β*1, leading to reduced gene transcription involved in vascular inflammation and remodeling, whereas this therapeutic effect is ineffective in endothelial cells, and the author hypothesizes that this is due to some problems with the binding of AGGF1 and integrin a5 receptors on the membrane of endothelial cells [29]. This is indeed a question worth exploring. In different diseases, different cells play completely different roles. After stent implantation, due to friction between the stent and the blood vessel wall, endothelial cells on the vessel wall are damaged or even disappear [46]. The barrier function of endothelial cells is to smooth blood flow through the vessel. When they are damaged, inflammatory factors and adhesion substances in the blood accumulate to form thrombi. At the same time, smooth muscle cells proliferate, which decreases the elasticity of the blood vessel wall and narrows the lumen. The role of GB on smooth muscle cells is precisely the right response to the condition of blood vessels after stent implantation.

GB effectively inhibits the Tgf*β*1/Smad signaling pathway, thereby reducing vascular remodeling and the recurrence of vascular stenosis. However, following LCA vascular injury in Tgf*β*1^−/−^ mice, the neointimal and vascular remodeling also occurred but was less severe than in wild-type mice, indicating that vascular remodeling and further restenosis are not only dependent on the Tgf*β*1/Smad signaling pathway. Previously, studies showed that the Notch signal pathway [47–49], micro-RNA [50–52], and AMPK/mTOR signaling pathway [53] all play varying roles in the process of vascular remodeling. Therefore, GB likely needs to be combined with other agents to effectively treat vascular remodeling.

Interestingly, our study found that the drug of pirfenidone capsules also has a protective function in vascular remodeling diseases apart from idiopathic pulmonary fibrosis.

## 5. Conclusions

In summary, the present study demonstrates the beneficial effect of GB on anti-inflammation, antifibrosis, antimacrophage infiltration, and vascular remodeling after vascular injury. Our study also demonstrates that the vascular remodeling and restenosis could not be completely abolished by suppressing Tgf*β*1. Additionally, we have broadened the indication of pirfenidone capsules and Yinxingneizhi Zhusheye for vascular remodeling disease. Finally, our data identify GB as a novel candidate drug for developing strategies to decrease vascular remodeling and prevent the recurrence of stenosis, which could potentially be used for the treatment of atherosclerosis and CAD.

## Data Availability

The data used to support the findings of this study are available from the corresponding authors upon request.

## Supplementary Materials

Supplementary Materials Additional statistical data are given in the supplementary material, including original western blot and statistical data.
