# Supplementary material for: Ginkgolide B Blocks Vascular Remodeling after Vascular Injury via Regulating Tgfβ1/Smad Signaling Pathway
Source: Cardiovasc Ther. 2023 Dec 13;2023:8848808. doi: 10.1155/2023/8848808 (PMC10732976; doi:10.1155/2023/8848808)
Supplement: Supplementary Materials — Additional statistical data are given in the supplementary material, including original western blot and statistical data. [file 8848808.f1.zip › Original data.pdf]

Figure 1

Figure a. The data of blood flow

Vehicle	RCA	131.17	96.596	101.416	112.432	137.285	123.774	127.15	124.69	113.171	132.089	124.289	119.862
	LCA	63.199	66.625	64.135	61.843	72.143	72.503	76.596	66.384	58.678	58.732	62.351	64.852
Ginkgolide I	RCA	125.959	112.965	108.148	106.149	106.384	137.865	135.943	122.978	118.652	126.491	114.682	124.354
	LCA	85.795	88.642	72.421	73.296	79.35	89.858	83.667	87.252	86.247	86.842	91.463	89.621

Figure d. The data of intima and media

Intima (um)

Vehicle	RCA	1.99	2.17	2.29	2.9
	LCA	121.65	135.93	133.04	108.57
Ginkgolide I	RCA	2.54	2.51	2.7	3.78
	LCA	16.32	15.31	21.17	19.8

media (um)

Vehicle	RCA	10.43	11.86	12.48	7.92
	LCA	27.04	26.54	31.25	31.8
Ginkgolide I	RCA	13.84	8.89	11.84	8.42
	LCA	22.32	18.88	18.33	17.31

The ratio of intima to media

Vehicle	RCA	0.1908	0.18297	0.18349	0.36616
	LCA	4.49889	5.1217	4.25728	3.41415
Ginkgolide I	RCA	0.18353	0.28234	0.22804	0.44893
	LCA	0.73118	0.81091	1.15494	1.14385

Figure e. The data of adventima

Vehicle	RCA	16.09	16.17	19.31	18.36
	LCA	46.21	45.34	43.77	42.32
Ginkgolide I	RCA	16.05	15.84	21.28	17.16
	LCA	38.96	33.48	25.07	30.53

Figure f. The data of Mcp1, Cd68, and α-Sma

		**Ct mean**				**ΔCT**			**ΔΔCT**			**2^(-ΔΔCT)**		
		Mcp1	Cd68	α-Sma	18S	Mcp1	Cd68	α-Sma	Mcp1	Cd68	α-Sma	Mcp1	Cd68	α-Sma
Vehicle	RCA	20.7909	28.0881	22.2104	11.3412	9.44974	28.0881	12.7607	0.25869	-0.08059	-0.16851	0.83585	1.05745	1.1239
		20.4484	28.0767	22.1515	11.3089	9.13958	28.0767	13.0119	-0.05146	-0.092	0.08276	1.03632	1.06585	0.94425
		20.9755	28.353	22.0406	11.5722	9.40326	28.353	12.6374	0.21221	0.18429	-0.29181	0.86321	0.88008	1.22417
		20.2184	28.157	22.0783	11.4468	8.77161	28.157	13.3067	-0.41944	-0.01169	0.37756	1.33741	1.00814	0.76974
	LCA	18.5909	25.2881	16.8104	11.4709	7.12001	25.2881	9.69038	-2.07104	-2.88059	-3.23878	4.20189	7.36453	9.43997
		18.2484	25.1767	16.1515	11.7232	6.52519	25.1767	9.62632	-2.66586	-2.992	-3.30285	6.34604	7.95578	9.86861
		18.0755	25.553	16.2406	11.1101	6.96534	25.553	9.27527	-2.22571	-2.61571	-3.65389	4.6774	6.12925	12.5872
		18.0184	25.357	16.2783	11.2954	6.72298	25.357	9.55536	-2.46807	-2.81169	-3.37381	5.53304	7.02107	10.3661
		20.767	28.1855	22.0176	11.6709	9.09602	28.1855	12.9216	-0.09502	0.01685	-0.00758	1.06808	0.98839	1.00527

Ginkgolide I	RCA	20.6472	28.0583	22.0761	11.9232	8.72396	28.0583	13.3522	-0.46709	-0.11039	0.42302	1.38232	1.07952	0.74586
		20.4624	28.2875	22.0369	11.3101	9.15231	28.2875	12.8846	-0.03874	0.1188	-0.04458	1.02721	0.92095	1.03138
		20.8405	28.1156	22.2396	11.4954	9.34507	28.1156	12.8946	0.15402	-0.05303	-0.0346	0.89874	1.03744	1.02427
	LCA	19.5675	27.6942	19.1574	11.739	7.8285	27.6942	11.3289	-1.36255	-0.47446	-1.6003	2.57139	1.3894	3.03207
		19.1099	27.019	19.3609	11.5584	7.55157	27.019	11.8094	-1.63947	-1.14965	-1.1198	3.11552	2.21859	2.17317
		19.5272	27.3682	19.0695	11.7629	7.76429	27.3682	11.3052	-1.42676	-0.80052	-1.62397	2.68843	1.74173	3.08221
		19.5772	27.0534	19.0243	11.3895	8.1877	27.0534	10.8366	-1.00335	-1.11528	-2.09253	2.00465	2.16637	4.26496

Figure 2

Figure a. The data of Tgfβ1 and p-Smad2/3 at protein level

Tgfβ1

Vehicle	RCA	0.99534	1.01215	0.99026	1.00226
	LCA	2.22267	2.10307	2.54604	2.24111
Ginkgolide I	RCA	1.0002	1.01013	1.084	1.03787
	LCA	1.64139	1.89068	2.07471	1.78369

p-Smad2/3

Vehicle	RCA	0.9074	0.87701	1.11013	1.10545
	LCA	1.71979	1.47045	1.65498	1.57078
Ginkgolide I	RCA	0.91219	0.9814	0.99825	0.98046
	LCA	1.31935	1.41568	1.23293	1.15409

Figure b. The data of pro-Tgfβ1 at mRNA level

		**Ct mean**		**ΔCT**	**ΔΔCT**	**2^(-ΔΔCT)**
		Pro-TGFb	18S	Pro-TGFb	Pro-TGFb	Pro-TGFb1
Vehicle	RCA	27.2733	11.3412	15.9321	0.09141	0.9386
		27.3013	11.3089	15.9924	0.15172	0.90018
		27.2639	11.5722	15.6917	-0.14897	1.10878
		27.1933	11.4468	15.7465	-0.09416	1.06745
	LCA	25.455	11.4709	13.984	-1.85664	3.62164
		25.4699	11.7232	13.7466	-2.09404	4.26943
		25.4093	11.1101	14.2992	-1.54146	2.9109
		25.4788	11.2954	14.1834	-1.65729	3.15423
Ginkgolide I	RCA	27.4103	11.6709	15.7394	-0.10132	1.07275
		27.2079	11.9232	15.2846	-0.55608	1.47027
		27.0743	11.3101	15.7642	-0.07647	1.05443
		27.1879	11.4954	15.6924	-0.14824	1.10822
	LCA	26.2201	11.739	14.4811	-1.35958	2.5661
		26.2827	11.5584	14.7243	-1.11639	2.16804
		26.5694	11.7629	14.8065	-1.03418	2.04795
		26.4827	11.3895	15.0932	-0.74751	1.67889

Figure c. The data of Tgfβ1 in serum

Vehicle	RCA	221.667	246.667	228.889	263.333
	LCA	524.444	530	522.222	520.556
Ginkgolide I	RCA	271.111	216.667	225	201.667
	LCA	321.667	328.333	334.444	322.778

Figure d. The data of Mcp1 in serum

Vehicle	RCA	24.7321	27.6786	29.8214	28.125
	LCA	174.107	175.982	172.5	167.232
Ginkgolide I	RCA	28.75	27.3214	29.8214	27.4107
	LCA	57.2321	58.3036	60.0893	56.5179

Figure e. The data of α-Sma in serum

Vehicle	RCA	12.5	10	15	17.5
	LCA	70	67.5	72.5	65
Ginkgolide I	RCA	12.5	17.5	15	7.5
	LCA	32.5	25	37.5	30

Figure 3

Figure a. The data of Tgfβ1 and p-Smad2/3 at protein level

Tgfβ1

	Control	1.092507	0.907493	0.932817	1.067183
	15 min	0.838109	0.802396	0.893833	0.923914
g/ml TGF-	30 min	1.038243	1.135569	1.598367	1.171451
	60 min	1.621646	1.832007	1.368692	1.371016

p-Smad2/3

	Control	0.994306	1.005694	0.959643	1.040357
	15 min	1.031931	1.2749	0.847227	0.903039
g/ml TGF-	30 min	1.752789	1.596808	1.34254	0.969243
	60 min	2.027706	2.103247	1.130079	1.303133

Figure b. The data of cell avalibity

	Control	1.3518	1.7948	1.7679	1.842	1.393	1.4711	1.6463	1.1065
	Buffer	1.3968	1.5298	1.7627	1.6706	1.5838	1.3586	1.4824	1.0218
	10	1.5102	1.4548	1.6307	1.48	1.3108	1.5419	1.2945	0.9358
	20	1.2761	1.3727	1.786	1.6935	1.9129	1.9097	1.2451	1.386
	40	1.2264	1.8499	1.9442	2.0633	2.1344	1.7617	1.3757	1.0448
kgolide B (	80	1.4801	1.7191	1.443	1.5478	1.8509	1.5645	1.5007	1.1141
	150	1.1223	0.8387	1.6596	1.3593	1.387	1.4987	1.103	1.1593
	300	1.2673	0.9448	0.8997	0.9113	0.9089	0.5793	0.6256	1.0004

Figure c. The data of Tgfβ1 in cell culture supernatant

Vehcle	Vehicle	201.6667	216.6667	238.8889	253.3333
	Ginkgolide	245.1111	226.6667	245	251.6667
TGF-β1	Vehicle	524.4444	503	542.2222	511.5556
	Ginkgolide	421.6667	528.3333	594.4444	542.7778

Figure d. The data of Tgfβ1 and p-Smad2/3 at protein level

Tgfβ1

Vehcle	Vehicle	1.012918	0.987082	1.120573	0.879427
	Ginkgolide	0.9396	0.787933	1.153329	1.636827

TGF-β1	Vehicle	1.522999	1.498041	2.432662	2.114956
	Ginkgolide	1.646056	1.563807	1.692235	1.84182

p-Smad2/3

Vehcle	Vehicle	1.015571	0.984429	1.039964	0.960036
	Ginkgolide	0.974496	0.940048	1.11686	1.394899
TGF-β1	Vehicle	2.003794	2.133331	1.77116	1.276684
	Ginkgolide	2.003851	1.982962	1.348072	1.279788

Figure 4

Figure a. The data of Tgfβ1 and p-Smad2/3 at protein level

Tgfβ1

	Control	1.163649	1.226027	0.802451	0.807873
	15 min	1.257037	1.310514	0.684004	0.587208
ıg/ml TGF-	30 min	1.963182	2.184753	1.044113	1.02278
	60 min	2.41533	2.439928	1.741596	1.624691

p-Smad2/3

	Control	0.954212	0.948061	0.996819	1.100908
	15 min	1.103877	1.28054	1.063404	1.124774
ıg/ml TGF-	30 min	1.451011	1.285736	1.232003	1.218916
	60 min	1.612328	1.72379	1.661117	1.517918

Figure b. The data of cell avalibity

	Control	1.1065	1.504	2.1713	2.1766	1.8444	1.2854	0.982	1.1596
	Buffer	1.296	1.7366	1.9462	1.8256	1.8605	1.2404	1.6489	1.0852
	10	1.296	1.7366	1.9462	1.8256	1.8605	1.2404	1.6489	1.0852
	20	1.2264	1.8499	1.9442	2.0633	2.1344	1.7617	1.3757	1.0448
	40	1.1668	1.6937	1.6527	1.2316	1.6442	1.3848	1.2357	1.1523
kgolide B (ı	80	0.9121	1.7271	1.4318	1.5991	1.7748	1.4986	1.4346	0.9348
	150	1.2638	1.6525	1.5676	1.4464	1.572	1.4593	1.1633	0.7631
	300	0.9437	1.009	0.9956	0.1944	1.1642	0.6375	0.7068	0.9353

Figure c. The data of Tgfβ1 in cell culture supernatant

Vehcle	Vehicle	221.6667	206.6667	218.8889	213.3333
	Ginkgolide	231.1111	236.6667	205	211.6667
TGF-β1	Vehicle	546.6667	524.4444	533.3333	537.2222
	Ginkgolide	316.1111	322.7778	328.8889	317.2222

Figure d. The data of Tgfβ1 and p-Smad2/3 at protein level

Tgfβ1

Vehcle	Vehicle	0.818255	1.181745	1.076977	0.923023
	Ginkgolide	1.236871	1.260823	1.044935	0.887961
TGF-β1	Vehicle	3.618315	3.333802	2.051978	2.256535
	Ginkgolide	1.712217	1.80189	1.070149	1.10207

p-Smad2/3

Vehcle	Vehicle	0.887252	1.112748	1.170818	0.829182
	Ginkgolide	0.87312	0.96138	1.12827	1.069485
TGF-β1	Vehicle	2.226798	1.987724	2.031137	2.025931

TGF-β1	Ginkgolide	1.435248	1.531969	1.623661	1.638297

Figure 5

Figure a. The data of Tgfβ1 and p-Smad2/3 at protein level tretment with PFD

Tgfβ1

PFD (ug/ml	0	1.009486	0.990514	0.943338	1.056662
	1	0.555839	0.631666	0.739412	0.79449
	10	0.498427	0.462268	0.624273	0.499332
	100	0.364838	0.352981	0.373995	0.404407

p-Smad2/3

PFD (ug/ml	0	1.058469	0.941531	0.967054	1.032946
	1	0.588904	0.641442	0.801707	0.795225
	10	0.483143	0.432381	0.669124	0.57902
	100	0.39694	0.329075	0.529624	0.52548

Figure b. The data of Tgfβ1 and p-Smad2/3 at protein level treatment with TGF-β1 and PFD

Tgfβ1

β1 (3 ng/ m	PFD (ug/ml	0	0.996551	1.003449	1.003259	0.996741
		1	0.666229	0.702653	1.000673	0.96585
		10	0.503885	0.383956	0.745794	0.80916
		100	0.471959	0.50641	0.605797	0.580193

p-Smad2/3

β1 (3 ng/ m	PFD (ug/ml	0	0.936541	1.063459	1.044264	0.955736
		1	0.669229	0.612251	0.954451	0.890535
		10	0.522732	0.52952	0.7578	0.76197
		100	0.526582	0.559596	0.690459	0.761485

Figure c. The data of Tgfβ1 and p-Smad2/3 at protein level treatment with TGF-β1, GB, and PFD

Tgfβ1

Vehcle	veh	veh	0.905853	0.893271	1.216988	0.983889
	PFD	veh	0.634216	0.521726	0.599246	0.49927
	veh	GB	0.868103	0.810708	1.036991	0.987838
	PFD	GB	0.531565	0.504154	0.622633	0.584279
TGF-β1	veh	veh	1.903944	1.815346	2.391713	1.729679
	PFD	veh	0.643721	0.586861	0.631875	0.545533
	veh	GB	1.379242	1.255847	1.743215	1.340995
	PFD	GB	0.565309	0.38793	0.676624	0.365032

p-Smad2/3

Vehcle	veh	veh	1.046654	1.083726	1.037209	0.832411
	PFD	veh	0.641161	0.515802	0.547502	0.461521
	veh	GB	0.943115	0.975732	0.829231	0.862849
	PFD	GB	0.692676	0.64935	0.58927	0.4192
TGF-β1	veh	veh	2.297986	1.989967	2.480509	1.674197
	PFD	veh	0.708209	0.658135	0.585147	0.400087
	veh	GB	1.930305	1.236689	1.563708	1.420138
	PFD	GB	0.55891	0.545854	0.51706	0.476443

Figure d. The data of Tgfβ1 in cell culture supernatant

veh	veh	225.5556	220.5556	231.6667	218.8889

Vehcle	PFD	veh	35	40	35	46.11111
	veh	GB	226.1111	226.6667	225	217.2222
	PFD	GB	29.44444	34.44444	40.55556	46.66667
TGF-β1	veh	veh	552.2222	546.6667	538.8889	542.7778
	PFD	veh	40.55556	45.55556	46.11111	30
	veh	GB	371.6667	378.3333	384.4444	372.7778
	PFD	GB	34.44444	39.44444	40	36.11111

Figure 6

Figure d. The data of blood flow in Tgfβ1 knockout mice

Vehicle	RCA	169.943	158.094	179.887	163.64	168.347	181.476	175.105	169.714
	LCA	95.184	95.02	99.453	112.059	105.966	112.339	106.489	112.059
PFD	RCA	144.769	189.873	233.073	146.369	180.062	124.87	161.008	121.681
	LCA	134.491	138.059	142.749	108.482	151.455	140.527	127.933	88.684
Ginkgolide I	RCA	179.069	128.902	146.25	148.944	136.192	182.389	183.271	185.421
	LCA	134.491	138.059	127.933	124.87	161.008	121.681	128.902	136.192

Figure e. The data of intima and media

Intima					
Vehicle	RCA	2.13	1.56	3.24	2.59
	LCA	43.23	41.34	45.32	40.05
PFD	RCA	2.58	2.12	1.99	3.43
	LCA	41.27	39.23	39.16	40.78
Ginkgolide I	RCA	3.21	3.01	2.12	2.09
	LCA	40.79	41.98	39.21	40.89
Media					
Vehicle	RCA	11.32	10.56	11.26	12.34
	LCA	22.32	24.51	21.36	21.68
PFD	RCA	12.86	11.21	10.46	9.98
	LCA	21.68	19.46	19.98	20.24
Ginkgolide I	RCA	8.97	12.35	13.26	10.89
	LCA	20.63	19.54	19.76	20.12

Figure e. The data of adventima

Vehicle	RCA	16.29	16.89	17.21	17.95
	LCA	41.26	40.32	40.86	40.59
PFD	RCA	18.03	17.98	17.88	17.26
	LCA	36.78	37.86	36.98	37.21
Ginkgolide I	RCA	19.21	18.76	17.21	17.03
	LCA	39.98	41.06	40.15	40.32

Figure g. The data of Mcp1, Cd68, and α-Sma

**Ct mean**				**ΔCT**			**ΔΔCT**			**2^(-ΔΔCT)**		
MCP1	CD68	α-Sma	18S	MCP1	CD68	α-Sma	MCP1	CD68	α-Sma	MCP1	CD68	α-Sma
20.394	17.471	26.406	17.695	2.698483	-0.22396	8.711048	-0.09152	0.069779	-0.08535	1.065492	0.952784	1.060945

**Vehicle**	**RCA**	20.518	17.245	26.604	17.678	2.839926	-0.43298	8.925987	0.049923	-0.13924	0.129589	0.965988	1.101324	0.914092
		20.568	17.359	26.485	17.661	2.907387	-0.30229	8.823481	0.117384	-0.00854	0.027082	0.921857	1.005938	0.981403
		20.345	17.415	26.356	17.631	2.714214	-0.21574	8.725077	-0.07579	0.078002	-0.07132	1.053937	0.947369	1.050679
	**LCA**	18.519	15.586	24.688	17.561	0.9583	-1.9742	7.127384	-1.8317	-1.68045	-1.66901	3.559569	3.205288	3.179972
		18.624	15.253	24.816	17.644	0.980518	-2.39105	7.172416	-1.80948	-2.09731	-1.62398	3.505169	4.279094	3.082246
		18.738	15.332	24.765	17.693	1.045141	-2.36051	7.071936	-1.74486	-2.06676	-1.72446	3.351626	4.189452	3.30457
		18.473	16.045	24.228	17.504	0.96974	-1.45863	6.724623	-1.82026	-1.16488	-2.07178	3.531454	2.242152	4.204037
**PFD**	**RCA**	20.345	17.415	26.356	17.631	2.714214	-0.21574	8.725077	-0.07579	0.078002	-0.07132	1.053937	0.947369	1.050679
		20.422	17.241	26.517	17.783	2.638599	-0.54192	8.733807	-0.1514	-0.24817	-0.06259	1.110649	1.187703	1.04434
		20.552	17.311	26.233	17.480	3.071314	-0.16933	8.753147	0.281312	0.124415	-0.04325	0.822843	0.917376	1.030433
		20.573	17.658	26.575	17.797	2.775976	-0.13902	8.77796	-0.01403	0.154719	-0.01844	1.00977	0.898307	1.012863
	**LCA**	18.806	15.232	24.646	17.610	1.19626	-2.37712	7.036379	-1.59374	-2.08337	-1.76002	3.018312	4.237975	3.387027
		18.368	15.988	24.616	17.702	0.665703	-1.7133	6.913739	-2.1243	-1.41956	-1.88266	4.359914	2.675036	3.687541
		20.281	17.124	26.340	19.357	0.923672	-2.23294	6.982626	-1.86633	-1.9392	-1.81377	3.646039	3.83493	3.515602
		20.253	17.243	26.461	19.353	0.900342	-2.10938	7.108598	-1.88966	-1.81564	-1.6878	3.70548	3.520154	3.22165
**inkgolide**	**RCA**	20.389	17.081	26.565	17.700	2.689037	-0.61939	8.864883	-0.10097	-0.32565	0.068485	1.072491	1.253227	0.953639
		20.352	17.436	26.861	17.738	2.613804	-0.30179	9.122833	-0.1762	-0.00804	0.326435	1.129903	1.00559	0.797505
		20.718	17.472	26.701	17.747	2.971094	-0.27504	8.953974	0.181092	0.018704	0.157576	0.882035	0.987119	0.89653
		20.456	17.365	26.425	17.587	2.868931	-0.22172	8.83773	0.078928	0.072021	0.041332	0.946761	0.951305	0.971757
	**LCA**	20.261	17.251	26.313	19.311	0.949858	-2.06046	7.00201	-1.84014	-1.76672	-1.79439	3.580457	3.402787	3.468683
		20.413	17.458	26.329	19.430	0.983816	-1.97188	6.899921	-1.80619	-1.67814	-1.89648	3.497166	3.200146	3.723031
		20.465	17.192	26.480	19.272	1.193096	-2.08013	7.208092	-1.59691	-1.78639	-1.58831	3.024939	3.4495	3.006962
		20.288	17.368	26.440	19.388	0.900097	-2.02042	7.051405	-1.88991	-1.72668	-1.74499	3.706109	3.309653	3.351933
