# Supplementary figures and images for: Ginkgolide B Blocks Vascular Remodeling after Vascular Injury via Regulating Tgfβ1/Smad Signaling Pathway
Source: Cardiovasc Ther. 2023 Dec 13;2023:8848808. doi: 10.1155/2023/8848808 (PMC10732976; doi:10.1155/2023/8848808)

Figure 2a

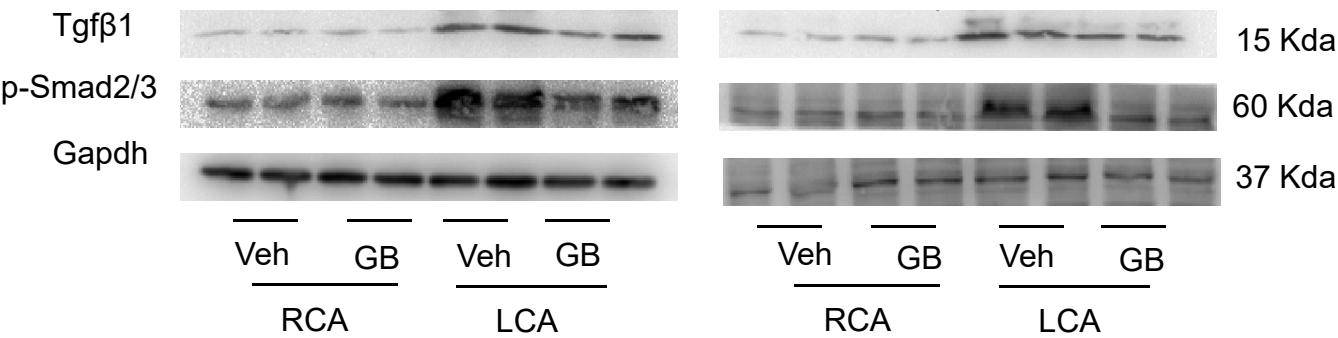

Figure 3a

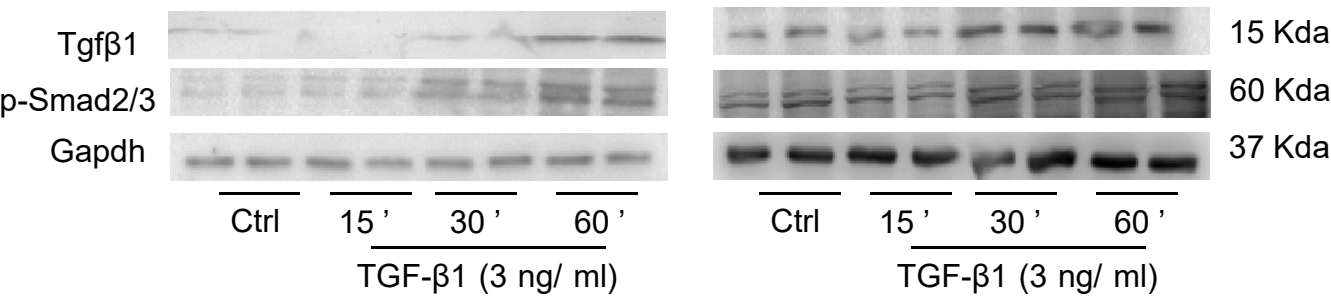

Figure 3b

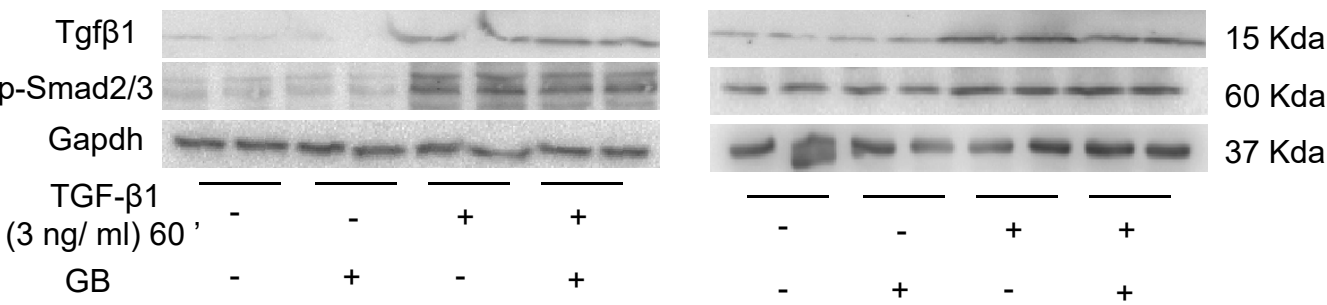

Figure 4a

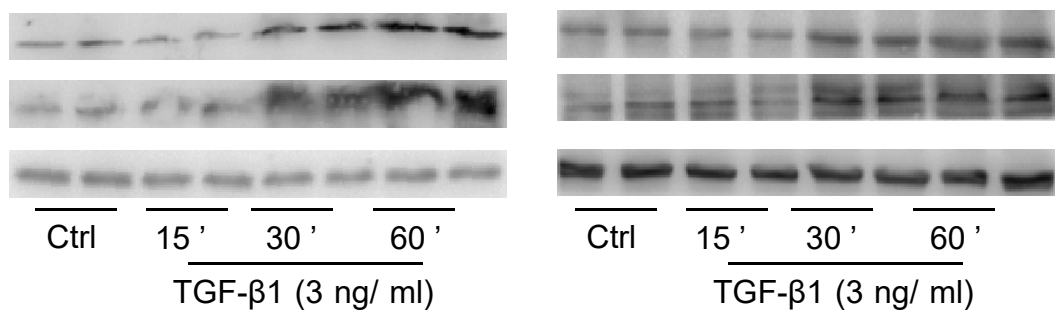

Figure 4d

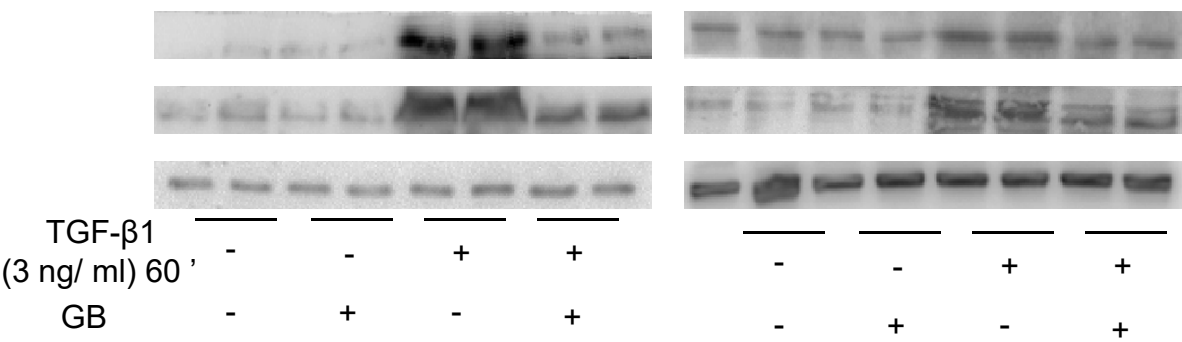

Figure 5a

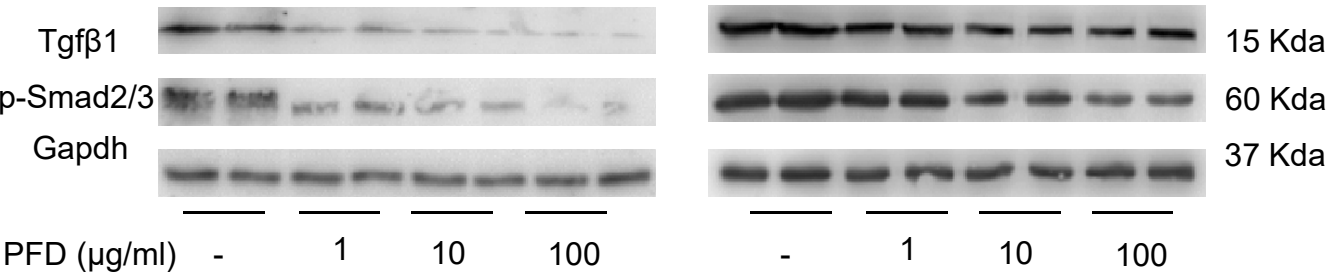

Figure 5b

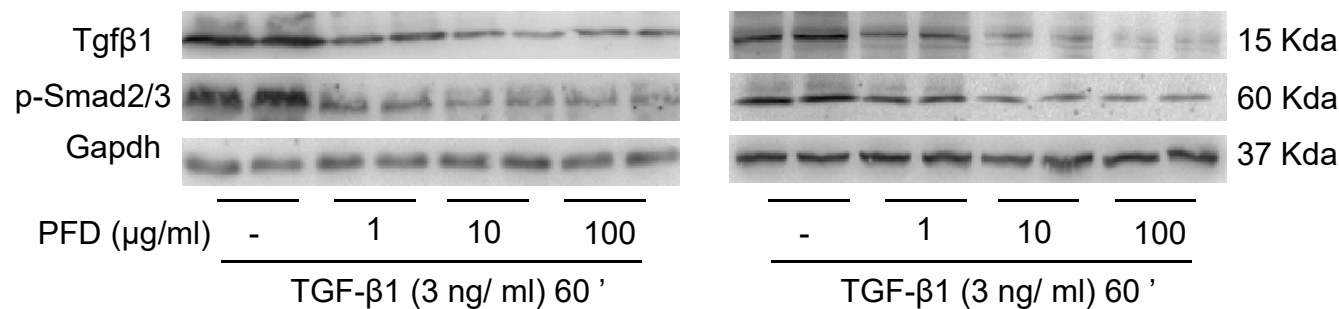

Figure 5c

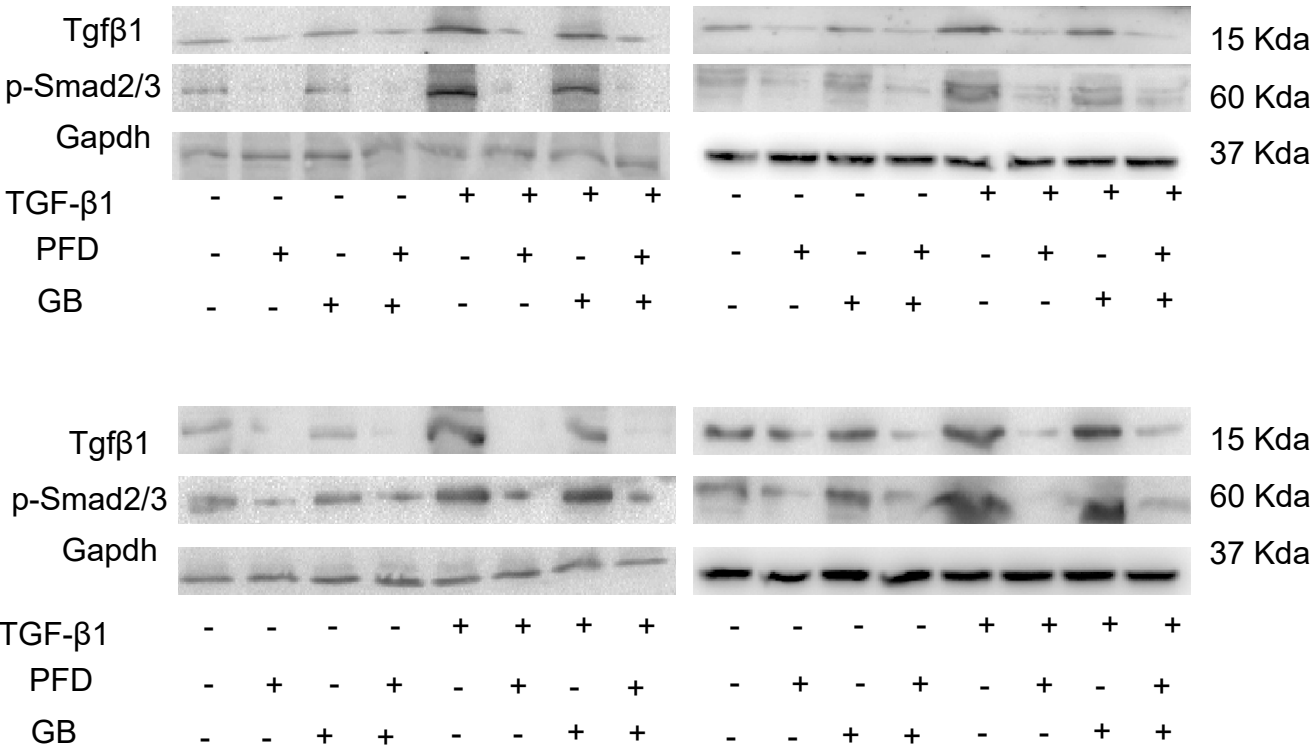

Supplement: Supplementary Materials — Additional statistical data are given in the supplementary material, including original western blot and statistical data. [file 8848808.f1.zip › Original western blot.pdf]
